# Association of *TMEM8B* and *SPAG8* with Mature Weight in Sheep

**DOI:** 10.3390/ani10122391

**Published:** 2020-12-15

**Authors:** Mehmet Ulas Cinar, Michelle R. Mousel, Maria K. Herndon, J. Bret Taylor, Stephen N. White

**Affiliations:** 1 Department of Veterinary Microbiology and Pathology, Washington State University, Pullman, WA 99164, USA; mehmet.cinar@wsu.edu (M.U.C.); mkmeyer@wsu.edu (M.K.H.); 2 Department of Animal Science, Faculty of Agriculture, Erciyes University, Kayseri 38039, Turkey; 3 Animal Disease Research Unit, Agricultural Research Service, U.S. Department of Agriculture, Pullman, WA 99164, USA; michelle.mousel@usda.gov; 4 School for Global Animal Health, Washington State University, Pullman, WA 99164, USA; 5 Range Sheep Production Efficiency Research Unit, Agricultural Research Service, U.S. Department of Agriculture, Dubois, ID 83423, USA; bret.taylor@usda.gov; 6 Center for Reproductive Biology, Washington State University, Pullman, WA 99164, USA

**Keywords:** SNP, ewe weight, U.S. sheep breeds, *NPR2*, *rs426272889*

## Abstract

**Simple Summary:**

Domestication and the subsequent selection of animals for either economic or morphological features can impact the legacy genome of a population in myriad ways. In sheep, the *rs426272889* single nucleotide polymorphism (SNP) was identified as the peak of a signature of selection. We examined phenotypic data and identified associations for the Transmembrane protein 8B (*TMEM8B*) *rs426272889* SNP and its genetically linked Sperm-associated antigen 8 (*SPAG8*) *rs160159557* SNP with ewe mature weight in four sheep breeds. These data provided the first production-relevant phenotypes, as well as the first organism-level (as opposed to cellular or tumor-derived) phenotypes, associated with *TMEM8B*, and in so doing, improved the annotation of this gene and genomic region by adding body weight implications. Once validated, these data can be applied in genetic or genomic selection aiming to achieve desired mature body weight.

**Abstract:**

Signature of selection studies have identified many genomic regions with known functional importance and some without verified functional roles. Multiple studies have identified Transmembrane protein 8B (*TMEM8B)* *rs426272889* as having been recently under extreme selection pressure in domesticated sheep, but no study has provided sheep phenotypic data clarifying a reason for extreme selection. We tested *rs426272889* for production trait association in 770 U.S. Rambouillet, Targhee, Polypay, and Suffolk sheep. *TMEM8B* *rs426272889* was associated with mature weight at 3 and 4 years (*p* < 0.05). This suggested selection for sheep growth and body size might explain the historical extreme selection pressure in this genomic region. We also tested Sperm-associated antigen 8 (*SPAG8*) *rs160159557* encoding a G493C substitution. While this variant was associated with mature weights at ages 3 and 4, it was not as strongly associated as *TMEM8B* *rs426272889.* Transmembrane protein 8B has little functional information except as an inhibitor of cancer cell proliferation. To our knowledge, this is the first study linking *TMEM8B* to whole organism growth and body size under standard conditions. Additional work will be necessary to identify the underlying functional variant(s). Once identified, such variants could be used to improve sheep production through selective breeding.

## 1. Introduction

Sheep were domesticated in the Fertile Crescent, where their wild relatives remain, 10,500 years before the present [1]. Domestication and artificial selection triggered positive selection for many traits in domestic species, reshaped livestock genomes, and left “footprints” called selection signatures [2]. These footprints can be used to identify genetic loci subjected to selection in the recent evolutionary past, including many involved with important livestock traits. The identification of such loci under selection may help to understand the molecular mechanisms involved in adaptation and may also be useful in identifying regions associated with important traits that are under selection [3]. Furthermore, the elucidation of such signatures could help identify genes with important but previously unappreciated roles and may also be useful in corroborating quantitative trait loci (QTL) mapping experiments in livestock [4].

Kijas et al. [2] used thousands of globally representative sheep to identify a small number of signatures of selection reflecting the recent evolutionary history of domestic sheep. Some included known color mutations and/or genes, but others were suggested to relate to production traits under strong selection. The SNP c.-303-1270C>T (*rs426272889*) on OAR2 was identified with strong evidence for a signature of selection in sheep. Although Kijas et al. [2] suggested Natriuretic peptide receptor-B (*NPR2*) as the nearest candidate gene based on genome annotation at the time, the SNP is located in the intron of Transmembrane protein 8B (*TMEM8B*) according to a more recent sheep genome assembly Oar_v3.1 [5]. Interestingly, the same OAR2 region in which Sperm-associated antigen 8 (*SPAG8*), Histidine triad nucleotide binding protein 2 (*HINT2*), *TMEM8B*, and *NPR2* are found was also identified as a signature of selection in various other sheep populations. Additional signatures of selection in domestic sheep have been identified in Chinese indigenous sheep breeds [6,7], Iranian sheep breeds [8,9], Mediterranean fat-tailed breeds [10], Ethiopian indigenous sheep breeds [11], worldwide sheep populations [12], Brazilian sheep breeds [4], sheep breeds from Russia [13], Western Pyrenees sheep [14], and Laticauda fat-tailed sheep from Italy [15]. Beside those findings, we also identified a missense SNP (*rs160159557*) in *SPAG8* gene, which is located near the reference SNP (*rs426272889*). Furthermore, to the best of our knowledge, no other research has been conducted on the association between *TMEM8B* and *SPAG8* polymorphisms and mature ewe bodyweight and production traits.

Therefore, the objectives of this study were to investigate the associations of *TMEM8B rs426272889* and the *SPAG8* Gly493Cys variant with individual ewe growth and lifetime production traits in US Polypay, Rambouillet, Targhee, and Suffolk sheep.

## 2. Material and Methods

### 2.1. Animals and Phenotyping

All animal care and use practices were reviewed by the Institutional Animal Care and Use Committees of Washington State University (protocols 3171, 4594) and the USDA, Range Sheep Production Efficiency Research Unit (approval 0703). Samples of distal ear tip or blood obtained via jugular venipuncture were collected from 770 Polypay, Rambouillet, Targhee, and Suffolk ewes at the U.S. Sheep Experiment Station (Dubois, ID, USA). Specifically, there were 188 Polypay, 359 Rambouillet, 168 Targhee, and 55 Suffolk ewes. Details of recorded phenotypes were previously given in Cinar et al. [16]. Briefly, traits examined included total weight of greasy fleece produced in a ewe’s life, lifetime number of lambs born, lifetime number of lambs born alive, lifetime number of lambs weaned, and total weight of lambs weaned over a lifetime as lifetime production traits. Individual traits were recorded, including birth weight, June bodyweight year of birth, adjusted bodyweight to a constant 120 days, weaning bodyweight, August/September bodyweight in the year born, average daily gain, bodyweight in April/May at 3 and 4 years old, bodyweight in September at 3 and 4 years old, udder score September at 3 and 4 years old, and subjective milk score 3 and 4 years old.

### 2.2. DNA Isolation and Genotyping

DNA was isolated from whole blood using the Invitrogen GeneCatcherTM gDNA 3-10 mL Blood Kit per manufacturers’ instructions (Invitrogen, Carlsbad, CA, USA). For ear tip samples, 3 to 5 mg of ear tissue had excess hair removed and then was placed in a Lysing Matrix A tube (MP Biomedical, Santa Ana, CA, USA) between two ceramic beads. DNA was extracted using the ChargeSwitch gDNA Micro Tissue Kit (Invitrogen, Carlsbad, CA, USA). Briefly, the tissue was allowed to rehydrate overnight at room temperature in lysis buffer (L15) and then processed with the Fast Prep FP120 machine (Thermo Savant, Carlsbad, CA, USA) followed by DNA isolation according to manufacturer’s protocol. Genotyping of single nucleotide polymorphisms included *TMEM8B* (*rs426272889*, OAR2: c.-303-1270C>T) and *SPAG8* (*rs160159557*, OAR2: p.Gly493Cys) using TaqMan SNP Genotyping Custom Assays (Invitrogen, Carlsbad, CA, USA). Briefly, genotyping was performed using allelic discrimination with primers (Invitrogen, Carlsbad, CA, USA) and hydrolysis probes (Invitrogen, Carlsbad, CA, USA) for rs426272889 (forward: CAAATTTCTTGGCCCTGACATTCAA, reverse: GCAGGAGAAGGTAAGGATGGAATC, VIC-TTGCTGCAAATGATGACAG, FAM-TTGCTGCAAATAATGACAG) and for rs160159557 (forward: GGAAAAATATCTTCCCTTGCCTGTCA, reverse: GCCTCCTCAGCCCTTAGAC, VIC-AGTAGTTCAACCCCCTCCAC, FAM- AGTAGTTCAACACCCTCCAC) with TaqMan Genotyping PCR Master Mix (Applied Biosystems, Foster City, CA, USA) and 50 ng of DNA in a final reaction of 10 μL in an ABI StepOnePlus Real-Time PCR System (Applied Biosystems, Foster City, CA, USA). Genotyping assays aimed for 95% genotyping rates in these data sets.

### 2.3. Statistical Analysis

The Haploview software package (Broad Institute, Cambridge, MA, USA) [17] was used to estimate and plot pairwise linkage disequilibrium (LD) measures (*D′* and *r*^2^). Phenotypic data were checked for normality before analyses with the univariate procedure of SAS v9.4 (SAS Institute, Cary, NC, USA). Genotype frequencies were calculated using the allele procedure of SAS v9.4. Individual animal data were analyzed with a mixed model using the mixed procedure of SAS v9.4. The reduced model included fixed effects of breed, year of birth (corresponded to age in years for this study), and genotype, with a random effect of sire nested within breed. Because Suffolks were monomorphic, they were not included in further analysis. In total, there were 238 sires in the data set, composed of 117 Rambouillet, 66 Polypay, and 55 Targhee. In addition, single breed analyses were run with similar models except no breed term and sire run without nesting. Genotypic comparisons were reported following Tukey-Kramer adjustment, and *p* ≤ 0.05 was considered significant.

## 3. Results

Descriptive statistics regarding measured phenotypes were given in Table S1. Both *r*^2^ and *D′* were estimated measures of linkage disequilibrium for the *TMEM8B* and *SPAG8* SNPs on OAR2 (Table S2). The *r*^2^ of 0.77 between SNPs and *D′* of 0.95 reveals the two SNPs are coinherited roughly 95% of the time. While Polypay, Rambouillet, and Targhee sheep breeds were found polymorphic for *TMEM8B rs426272889* and *SPAG8 rs160159557* SNPs, the Suffolk breed was monomorphic for these loci (Tables S3 and S4), so they were not used for further analysis. Three significant associations were observed between *rs426272889* SNP and ewe mature weights in spring at age 3, fall at age 3, and spring at age 4 years in multibreed analysis (Table 1). The TT (thymine homozygote) individuals were significantly heavier compared with CC (cytosine homozygote) animals (Table 1), and for fall at age 3, the difference was highly significant (*p* < 0.01). Three significant associations also were observed between *rs160159557* SNP and ewe mature weights in spring at age 3, fall at age 3, and spring at age 4 years (Table 2). Similar associations were also observed in Rambouillet single-breed analysis (Tables S5 and S6), except for spring weight at 3 years of age, which showed a trend toward significance for *TMEM8B rs426272889* (*p* = 0.058) and for *SPAG8 rs160159557* (*p* = 0.087). However, no additional associations were identified between *rs42627288* or *rs160159557* SNPs with other measured individual and lifetime ewe production and prolificacy traits in Idaho sheep populations (*p* > 0.05).

## 4. Discussion

An important open question for many identified signatures of selection is which traits may drive selection at those loci [2]. The methods used to identify selection signatures can pinpoint recent large, evolutionary differences in allele frequencies and other data to demonstrate historical selection, but the connection to biological function requires complementary phenotypic data. The use of phenotypic data from large numbers of sheep for trait association can help fill this need. Here, we identified association between *TMEM8B* and *SPAG8* SNPs and ewe mature body weight (Table 1 and Table 2). When significant associations are found, multibreed findings are superior because linkage disequilibrium is likely to be shorter in across-breed analysis, which makes detection of association more difficult [18,19]. The Rambouillet, Polypay, and Targhee breeds all have substantial contributions from Rambouillet ancestors, and likely have common mutations segregating. The across-breed analysis thus leveraged data from all these breeds to achieve the best overall results in Table 1 and Table 2. The data set included the largest number of Rambouillet sheep, and single-breed analysis in Rambouillets showed some, but not all, of the associations observed in the multibreed analysis (Tables S5 and S6). While in single-breed analysis, the Polypay and Targhee breeds did not show any association of either *TMEM8B rs426272889* or *SPAG8 rs160159557* with mature weight, the similar trends in their data and improved power from a larger number of animals in the combined data set permitted detection of these associations (Table 1 and Table 2). Furthermore, the use of a large, multibreed sheep population enabled robust estimation of genotypic means.

An interesting question regards the timing of association with respect to sheep age. The lack of statistically significant association between *TMEM8B* and *SPAG8* SNPs and body weights at younger ages might have occurred due to smaller body size differences at younger ages. The Animal QTL database exhibits few rare overlapping associated loci even for similar traits across different ages, suggesting this is not an unusual occurrence [20]. Marker-assisted selection can substantially increase the accuracy of selection in breeding programs for traits that are difficult and/or expensive to measure after a certain age, such as mature body weight [19].

TMEM8B is a tumor metastasis inhibitor formerly known as nasopharyngeal carcinoma associated 6 (NGX6) and was originally isolated from nasopharyngeal carcinoma [21,22] and colon cancer [23]. The *TMEM8B* gene acts to inhibit metastasis and restrains proliferation by arresting cell cycle progression at the G0/G1 phase of colon cancer cells in vitro [24]. Although transmembrane protein 8c (TMEM8C) and TMEM8B are close relatives and TMEM8C functions as a muscle-specific membrane protein in myoblast fusion, expression of TMEM8B protein is not muscle-specific [25]. To the best of our knowledge, this is the first report of any genetic association with *TMEM8B* in any livestock species.

SPAG8 was previously designated as hSMP-1 or HSD-1 and originally isolated from a human testis expression library using antisperm antibodies obtained from the serum of an infertile woman [26]. SPAG8 has been localized in spermatids and in the head and tail of sperm in rat testis, suggesting a role for SPAG8 in germ cell differentiation [26]. Although no association has been identified between *SPAG8* and any growth trait in humans or animals, loci near *TMEM8B* and *SPAG8* have been suggested as signatures of selection both in sheep and cattle [2,27,28,29].

The presents results may suggest one or more additional genetic variants in linkage disequilibrium with the reference SNP in *TMEM8B* that could be more functionally important in body size determination. The one other potential gene that is within 50 kb of the reference *TMEM8B* SNP is the natriuretic peptide receptor-B (*NPR2*) suggested by Kijas et al. [2]. C-type natriuretic peptide (CNP), which is a member of the natriuretic peptide family, binds to a homodimeric transmembrane receptor named NPR2. Previous literature has shown that CNP/NPR2 signaling is an important regulator of skeletal growth and CNP overexpression cause excessive growth in mice [30], while defects or mutations of the *NPR2* gene lead to impairment of skeletal development and short stature [31,32]. Given that sheep have been under selection for body size in many contexts, it is possible that an underlying mutation in linkage disequilibrium with *TMEM8B rs426272889* could mediate effects through *TMEM8B*, *NPR2*, or both. For example, U.S. Suffolk sheep have been heavily selected for increased weaning weights and have developed large frames. The Suffolk sheep in this study were homozygous TT for both *rs426272889* and *rs160159557*, which could have resulted from selection for growth rates and body size [33].

Many signatures of selection have been detected in various livestock species, including sheep [2,4,6,7,8,9,10,11,12,13,14]. An abundance of literature on detection of selection signatures may help in better understanding of genetic mechanisms affecting phenotypic differentiation in livestock [2,34,35]. However, follow-up studies integrating selection signature analysis with phenotype association analysis are useful to better understand the genetic mechanisms of functional variants [2,34,35].

## 5. Conclusions

Here, we report the association of sheep selection signature *TMEM8B rs426272889* SNP with ewe mature weight in a multibreed sheep population (Table 1). Validation of selection signatures may provide a basis for a better understanding of the forces driving artificial selection and will help in the design of more efficient livestock breeding programs. To the best of our knowledge, this is the first example of association between a widely observed selection signature near *rs426272889* SNP with direct phenotype measurements—in this case, mature body weight—in a large panel of sheep breeds. In conclusion, the present study revealed that *TMEM8B* and *SPAG8* are significantly associated with 3- and 4-year-old ewe mature weight, suggesting that *rs426272889* and *rs160159557* genotypes may serve as candidate genetic variants for marker-assisted selection programs in terms of lower mature body weight in sheep.

## Figures and Tables

**Table 1:** Association of *TMEM8B rs426272889* with ewe mature weights showing adjusted means and standard errors from Rambouillet, Polypay, and Targhee sheep.

Traits	CC (*n* = 182)	CT (*n* = 313)	TT (*n* = 185)
Weight in April/May of 3 years old (kg)	70.33 ± 0.90 ^a^	70.77 ± 0.78 ^a^	72.82 ± 0.90 ^b^
Weight in September of 3 years old (kg)	78.03 ± 0.81 ^a^	79.05 ± 0.71 ^a,b^	80.63 ± 0.81 ^b^
Weight in April/May of 4 years old (kg)	71.67 ± 0.91 ^a^	73.77 ± 0.80 ^b^	74.43 ± 0.91 ^b^

Genotype groups with differing letter designations were significantly different (*p* ≤ 0.05). CC: cytosine homozygote; CT: cytosine-thymine heterozygote; TT: thymine homozygote.

**Table 2:** Association of *SPAG8 rs160159557* (G493C) association with ewe mature weights, showing adjusted means and standard errors from Rambouillet, Polypay, and Targhee sheep.

Traits	GG (*n* = 176)	GT (*n* = 292)	TT (*n* = 205)
Weight in April/May of 3 years old (kg)	70.15 ± 0.91 ^a^	70.69 ± 0.81 ^a^	72.67 ± 0.89 ^b^
Weight in September of 3 years old (kg)	77.90 ± 0.81 ^a^	78.90 ± 0.73 ^a,b^	80.45 ± 0.80 ^b^
Weight in April/May of 4 years old (kg)	71.53 ± 0.93 ^a^	73.76 ± 0.83 ^b^	74.43 ± 0.91 ^b^

Genotype groups with differing letter designations were significantly different (*p* ≤ 0.05). GG: guanine homozygote; GT: guanine-thymine heterozygote; TT: thymine homozygote.

## Supplementary Materials

The following are available online at https://www.mdpi.com/2076-2615/10/12/2391/s1, Table S1: Descriptive statistics for traits measured: abbreviations, number of animals per trait (n), mean, standard error (se), minimum (Min.), and maximum (Max.) values, Table S2: Summary of analyzed SNPs and average linkage disequilibrium on OAR2, Table S3: TMEM8B genotype frequency data in various breeds of sheep from a single location, Table S4: SPAG8 genotype frequency data in various breeds of sheep from a single location, Table S5: Association of TMEM8B rs426272889 with ewe mature weights showing adjusted means and standard errors in Rambouillet sheep, Table S6: Association of SPAG8 rs160159557 (G493C) association with ewe mature weights, showing adjusted means and standard errors in Rambouillet sheep.

## Software and Data Repository Resources

Data were submitted to Open Science Framework (https:osf.io) from the Center for Open Science, and are available under doi:10.17605/OSF.IO/763KU.
