# Supplementary material for: Association of TMEM8B and SPAG8 with Mature Weight in Sheep
Source: Animals (Basel). 2020 Dec 15;10(12):2391. doi: 10.3390/ani10122391 (PMC7765121; doi:10.3390/ani10122391)
Supplement: Supplementary file 1 [file animals-10-02391-s001.pdf]

animals

Supplementary Material

Association of *TMEM8B* and *SPAG8* with mature weight in sheep

Mehmet Ulas Cinar [1,2], Michelle R. Mousel [3,4], Maria K. Herndon [1], J. Bret Taylor [5] and Stephen N. White [1,3,6,*]

1 Department of Veterinary Microbiology and Pathology, Washington State University, Pullman, WA 99164, USA; mehmet.cinar@wsu.edu (M.U.C.), mkmeyer@wsu.edu (M.K.H.)
2 Erciyes University, Faculty of Agriculture, Department of Animal Science, Kayseri 38039, Turkey
3 Animal Disease Research Unit, Agricultural Research Service, U.S. Department of Agriculture, Pullman, WA 99164, USA; michelle.mousel@usda.gov
4 School for Global Animal Health, Washington State University, Pullman, WA 99164, USA
5 Range Sheep Production Efficiency Research Unit, Agricultural Research Service, U.S. Department of Agriculture, Dubois, ID 83423, USA; bret.taylor@usda.gov
6 Center for Reproductive Biology, Washington State University, Pullman, WA 99164, USA
* Correspondence: stephen.white@usda.gov

Received: 11 November 2020; Accepted: 13 December 2020; Published: date

Table S1. Descriptive statistics for traits measured: abbreviations, number of animals per trait (n), mean, standard error (se), minimum (Min.), and maximum (Max.) values.

Type of Recording	Trait	Abbreviation	Mean	SE	Min	Max
Individual	Birth weight (kg)	BWT	4.88	0.75	2.67	7.75
	June weight year of birth (kg)	JWT	27.32	3.19	16.78	40.82
	Adjusted weight to a constant 120 days (kg)	WT120	38.16	4.07	28.12	57.15
	Weaning weight (kg)	WWT	37.07	4.68	29.48	68.03
	August/September weight in the year born (kg)	ASWT	42.28	4.74	29.48	68.03
	Average daily gain (kg)	ADG	0.27	0.03	0.19	0.43
	Weight in April/May of 3 years old (kg)	SPWT3	71.22	9.02	50.80	95.25
	Weight in September of 3 years old (kg)	FAWT3	79.07	8.19	57.60	113.39
	Weight in April/May of 4 years old (kg)	SPWT4	72.13	9.38	48.53	109.76
	Weight in September of 4 years old (kg)	FAWT4	82.01	8.56	53.97	114.75
	Udder score September of 3 years old ewe	UDDER3			Bad	Good
	Udder score September of 4 years old ewe	UDDER4			Bad	Good
	Subjective milk score 3 years old ewe	MILK3			0	5
	Subjective milk score 4 years old ewe	MILK4			0	5
Lifetime	Total weight of greasy fleece produced in a ewe's life (kg)	LFLWT	25.60	7.35	2.63	44.45
	Lifetime number of lambs born (count)	LLB	11.89	3.40	4.0	23.0
	Lifetime number of lambs born alive (count)	LLBA	11.06	3.20	3.0	21.0
	Lifetime number of lambs weaned (count)	LLW	8.93	2.63	2.0	17.0
	Total weight of lambs weaned over a lifetime (kg)	LLWW	317.43	93.30	56.69	596.47
	Age	Age	6.65	1.07	5.0	9.0

Supplementary Table S2. Summary of analyzed SNPs and average linkage disequilibrium on OAR2.

SNP1 (*TMEM8B*)	SNP2 (*SPAG8*)	D'	r^2	Distance (bp)
OAR2:52409379	OAR2:52426470	0.953	0.768	17091

Table S3. *TMEM8B* genotype frequency data in various breeds of sheep from a single location.

		Breeds				
		Polypay	Rambouillet	Targhee	Suffolk	Overall
Polymorphism	Genotypes	n	n	n	n	n
TMEM8B *rs426272889* c.-303-1270C>T	CC	58	92	32	0	182
	CT	80	168	65	0	313
	TT	47	81	57	55	240
	Total	185	341	154	55	735

n: number of individuals. CC: cytosine homozygote; CT: cytosine-thymine heterozygote; TT: thymine homozygote.

Table S4. *SPAG8* genotype frequency data in various breeds of sheep from a single location.

		Breeds				
		Polypay	Rambouillet	Targhee	Suffolk	Overall
Polymorphism	Genotypes	n	n	n	n	n
SPAG8 *rs160159557* g.52426470G>T	GG	55	92	29	0	176
	GT	61	161	70	0	292
	TT	49	87	69	55	260
	Total	165	340	168	55	728

n: number of individuals. GG: guanine homozygote; GT: guanine-thymine heterozygote; TT: thymine homozygote.

Supplementary Table S5. Association of *TMEM8B rs426272889* with ewe mature weights showing adjusted means and standard errors in Rambouillet sheep.

Traits	CC (n = 92)	CT (n = 168)	TT (n = 81)
Weight in September at 3 years (kg)	76.08 ± 0.96 [a]	78.34 ± 0.80 [a,b]	80.01 ± 1.01 [b]
Weight in April/May at 4 years (kg)	68.92 ± 1.06 [a]	71.68 ± 0.89[b]	71.93 ± 1.12 [a,b]

Genotype groups with differing letter designations were significantly different ($p \leq 0.05$).

Supplementary Table S6. Association of *SPAG8 rs160159557* (G493C) association with ewe mature weights, showing adjusted means and standard errors in Rambouillet sheep.

Traits	GG (n = 92)	GT (n = 161)	TT (n = 87)
Weight in September at 3 years (kg)	76.32 ± 0.95 [a]	78.28 ± 0.83 [a,b]	79.97 ± 1.00 [b]
Weight in April/May at 4 years (kg)	69.13 ± 1.07 [a]	71.86 ± 0.94 [b]	72.55 ± 1.14 [b]

Genotype groups with differing letter designations were significantly different ($p \leq 0.05$).
